# Source analysis and health risk assessment of polycyclic aromatic hydrocarbon (PAHs) in total suspended particulate matter (TSP) from Bengbu, China

**DOI:** 10.1038/s41598-024-55695-1

**Published:** 2024-03-01

**Authors:** Danchen Wu, Liu Chen, Zhijing Ma, Dalin Zhou, Le Fu, Mengmeng Liu, Tianer Zhang, Jing Yang, Quan Zhen

**Affiliations:** 1 School of Public Health, Bengbu Medical College, Bengbu, 233030 People’s Republic of China; 2 School of Laboratory Medicine, Bengbu Medical College, Bengbu, 233030 People’s Republic of China; 3 Fuyang Cancer Hospital, Fuyang, 236010 People’s Republic of China; 4 Xinchang Center for Disease Control and Prevention, Xinchang, 312599 People’s Republic of China

**Keywords:** Climate sciences, Environmental sciences

## Abstract

The polycyclic aromatic hydrocarbon (PAH) concentrations in total suspended particulate matter (TSP) samples collected from October, 2021 to September, 2022 were analyzed to clarify the pollution characteristics and sources of 16 PAHs in the atmospheric TSP in Bengbu City. The ρ(PAHs) concentrations ranged from 1.71 to 43.85 ng/m^3^ and higher concentrations were detected in winter, followed by spring, autumn, and summer. The positive matrix factorization analysis revealed that, in spring and summer, PAH pollution was caused mainly by industrial emissions, gasoline and diesel fuel combustion, whereas in autumn and winter, it was coal, biomass and natural gas combustion. The cluster and potential source factor analyses showed that long-range transport was a significant factor. During spring, autumn, and winter, the northern and northwestern regions had a significant impact, whereas the coastal area south of Bengbu had the greatest influence in summer. The health risk assessment revealed that the annual total carcinogenic equivalent concentration values for PAHs varied from 0.0159 to 7.437 ng/m^3^, which was classified as moderate. Furthermore, the annual incremental lifetime cancer risk values ranged from 1.431 × 10^−4^ to 3.671 × 10^−3^ for adults and from 6.823 × 10^−5^ to 1.749 × 10^−3^ for children, which were higher than the standard.

## Introduction

Polycyclic aromatic hydrocarbons (PAHs) are hydrocarbons with more than two benzene rings in the molecule, they are chemically stable, and are widely distributed in the environment. Studies have shown that long-term exposure to high PAH concentrations in the atmosphere can cause eye irritation, nausea, vomiting, diarrhea, and other symptoms^1^. They can enter the alveolar tissues via respiration and are deeply absorbed into the blood circulation, which may lead to increased morbidity and mortality from respiratory and cardiovascular diseases^2^. They also have potent carcinogenic, teratogenic, and mutagenic effects^3^. The US EPA lists 16 monomeric PAHs as priorities for control, seven of which are classified as potentially carcinogenic^4^.

Atmospheric PAHs mainly originate from the incomplete combustion of fossil fuels and wood, vehicle, and household emissions^5^. The identification of pollution sources, potential source areas, and health risks is essential for effective control of PAH emissions from different sources. The diagnostic ratios (DR) method can qualitatively identify pollution sources based on the ratio of PAHs with different number of rings and the distribution of PAHs in the parent nucleus^6^. The method was used by Ravindra et al.^7^ to indicate that fossil fuel combustion is the main source of atmospheric PAHs in a Belgian region and by Wang et al.^8^ to show that the major sources of atmospheric PAHs in the city of Dalian, China, were coal combustion and traffic emissions. The positive matrix factorization (PMF) method has also been widely used to quantitatively identify particulate-related PAH sources^9^, e. g. Bowen He et al.^10^ concluded that in the spring season, coal and biomass combustion (32.19%) and traffic emissions (28.06%) were the main contributors to PAH pollution in Chengde city, China, followed by crude oil and volatile oil spills (23.91%), with industrial sources (15.84%) making the lowest contribution. Moeinaddini et al.^11^ used the PMF method to investigate the sources of atmospheric PAHs in Iran and identified five PAH source factors, which were diesel fuel (56.3%), petrol (15.5%), wood burning and incineration (13%), industry (9.2%), and road soil particles (6.0%). Therefore, the diagnostic ratios method combined with the PMF method can be used to confirm the results from each method on its own when identifying the traced PAH sources.

Polycyclic aromatic hydrocarbons can be transported atmospherically over long distances^12^. Therefore, the spatial origin of pollutants is important. Liu et al.^13^ in their study on air pollution in the Shanghai region used a cluster analysis of the backward trajectory of air masses combined with the potential source contribution function (PSCF). The results showed that winter winds had a greater impact on air quality in the Shanghai region and that the areas making the largest pollution contributions were mainly concentrated in the northern part of China. Source area identification based on cluster analysis and the PSCF method is a prerequisite for improving pollutant emission management. However, it has been rarely used in previous studies^14^. In this study, these two methods were used to evaluate the pollutant transport pathways and major pollutant source areas of PAHs in Bengbu City, China.

China has the highest PAH emissions in the world. Satellite retrieval results show that the PAH emission density is highest in East China and that the PAH industrial emission density in the Yangtze River Delta region is as high as 10.78 kg/km^2^, much higher than the national average (3.05 kg/km^2^)^15^. Bengbu is located in the western part of the Yangtze River Delta region. It straddles both sides of the Huai River, is a land and water transport hub, a junction of the Beijing-Shanghai and Huainan railways, and an important city in northern Anhui Province^16^. The acceleration in urbanization has meant that air quality in Bengbu is becoming an important issue^17^. Environmental monitoring data in recent years has shown that the air quality in Bengbu is ranked at the bottom of the list of cities and municipalities in Anhui Province and is even worse than in the provincial capital of Hefei^18^. Some scientists are concerned about PAH pollution in the Yangtze River Delta region and have investigated the PAH air pollution status in Shanghai, Hefei, Hangzhou, and Nanjing^13,19–22^. Most of these studies focused on large cities with prosperous economies and very few small and medium-sized cities that occupy more territory have been investigated. As far as can be ascertained, no investigation of airborne PAHs has been conducted in Bengbu City. Previous studies mainly focused on investigating the local PAH pollution status rather than the impact of the long-range transport of air masses on PAHs from a regional perspective. Furthermore, previous studies concentrated on PAHs in PM_2.5_ or PM_10_, and there have been few studies on PAHs in total suspended particulate matter (TSP). Total suspended particulate matter can enter the human body via the respiratory tract, which may lead to an underestimation of the public health risk posed by PAHs in PM_10_ and PM_2.5_. In addition, previous health risk assessments were mostly been carried out using total carcinogenic equivalent concentration values and annual incremental lifetime carcinogenic risk values and only a small number of risk assessments concentrated on the different sources of PAHs.

Therefore, this study undertook sentinel monitoring of PAHs in TSP samples from Bengbu City over one year with the following objectives: (1) to uncover the changes in PAH concentration levels, seasonal trends, and distribution characteristics of PAHs with different ring counts; (2) to explore the possible sources and contributions made by PAHs using the characteristic ratio method and a PMF analysis, respectively; (3) to analyze the influence of air masses that have been transported from remote regions on the PAH characteristics in Bengbu City using the backward trajectory clustering method and the potential source contribution model; and (4) to assess the health risk to adults and children by calculating the population carcinogenic equivalent concentration and the lifetime carcinogenic risk. This study provides first-hand data that can be used to improve understanding about airborne PAHs pollution and its impact on human health in Bengbu City and a theoretical basis for the formulation of local air pollution control strategies. It also supplies basic data and technical support for the joint prevention and control of PAHs pollution in the western Yangtze River Delta region.

## Materials and methods

### Sampling sites and sample collection

The sampling point was the rooftop of the teaching building at Bengbu Medical College (117.433°E, 32.908°N) and the sampling period was from October, 2021 to September, 2022 with a collection period of 10 days at the beginning of each month. The TSP samples were collected according to National Standard GB/T39193-2020 “Determination of Ambient Air Particulate Matter Quality Concentration by Weight”, using a high-flow particulate sampler (Qingdao Laoying Environmental Science and Technology Co. Ltd, Quingdao, China). The sampling flow rate was 1.05 m^3^/min; 23 hourly samples were collected per day; and the sampling time and volume were recorded. The samples were sealed, returned to the laboratory, and stored in a refrigerator at – 20 ℃ in the dark.

### Sample processing

The method in “Determination of polycyclic aromatic hydrocarbons in ambient air and exhaust gas phase and particulate matter by gas chromatography-mass spectrometry (HJ 646–2013)” was used with slight modification. A 1/32 filter membrane was placed in a 20 mL sample bottle and a dichloromethane: methanol (methanol 2:1, V/V) solution was added until the filter membrane was completely immersed. The sample bottle was ultrasonically extracted twice for 10 min using an ultrasonic cleaner. The extracted solution was filtered through glass wool into a conical flask using a sterilized disposable dropper and the conical flask was placed on water bath nitrogen blowing apparatus to concentrate the solution. Then n-hexane and the internal standard was added and the solution was transferred to a gas chromatography vial. The analytical instrument was a gas chromatography-mass spectrometer (Agilent 7890A tandem gas chromatograph with an Agilent 5975C mass detector; Agilent Technologies, Santa Clara, CA, USA) equipped with an HP-5MS (30 m × 0.25 mm × 0.25 μm) fused silica capillary column (J&W Scientific, Folsom, CA, USA) and the carrier gas was high purity helium^23^.

A GC–MS instrument was used to detect 16 particulate PAH monomers^24^, which were naphthalene (Nap 2 ring), acenaphthylene (Acy 3 ring), acenaphthene (Ace 3 ring), fluorene (Flu 3ring), phenanthrene (Phe 3 ring), anthracene (An 3 ring), fluoranthene (Flt 4 ring), pyrene (Pyr 4 ring), benzo[a]anthracene (BaA 4 ring), Chr (Chr 4 ring), benzo[b]fluoranthene (BbF 5 ring), benzo[k]fluoranthene (BkF 5 ring), benzo[a]pyrene (BaP 5 ring), indeno[1,2,3-cd] pyrene (IcP 6 ring), dibenzo[a,h]anthracene (DBA 6 ring), and benzo[g,h,i]pyrene (BjhiP 6 ring).

### Quality assurance and quality control

The atmospheric samplers undergo routine flow rate calibration to ensure precise sampling. Throughout the experimental procedures, meticulous quality control and assurance were maintained through the utilization of parallel samples, method blanks, program blanks, and standard spiked recovery samples during the testing phase. In the detection process, the correlation coefficient for the standard curve of the 16 PAHs exceeded 0.99. The relative deviation of PAHs in parallel samples was rigorously controlled below 15%, accompanied by a sample repeatability of 10%. The specified PAHs were absent in the program blank. Deuterated PAHs (naphthalene-d8, acenaphthene-d10, phenanthrene-d10, chrysene-d12, and perylene-d10, o2si smart solutions, USA) were added to the samples as recovery surrogates. The PAH recoveries of the surrogates and the 16 PAH standard-spiked matrix recoveries were all within the acceptable range of 78.32% to 121.40%. The method detection limits are given in Table 1.

**Table 1:** Method detection limit (MDL) for individual PAHs.

Kinds	NaP	Acy	Ace	Flu	Phe	Ant	Fla	Pyr	BaA	Chr	BbF	BkF	BaP	IcdP	DBahA	BghiP
MDL (ng/m^3^)	0.026	0.008	0.008	0.011	0.015	0.031	0.014	0.033	0.029	0.030	0.021	0.013	0.015	0.015	0.003	0.009

### Source apportionment techniques

#### Diagnostic ratios

Four characteristic ratios^10^: Flu/ (Flu + Pyr), BaA/ (BaA + Chr), Ant/ (Ant + Phe), and InP/ (InP + BghiP), were calculated to determine the principal sources of PAHs in Bengbu City over the seasons of the year.

#### Positive matrix factorization modeling

In addition to the quantitative analysis of the diagnostic ratios method, this study also used the positive definite matrix factorization (PMF) analysis program EPA PMF5.0 to analyze the PAH sources and quantify the contributions made by different emission sources^25–27^, the basic principle of which is as follows^28^: assuming that *X* is the matrix of pollutant concentrations in a receptor sample, then *X* = *n* × *m*, where *n* is the number of samples and *m* is the number of chemical components. *X* can be decomposed into a contribution ratio matrix *G*, a factor component spectral loading matrix *F*, and a residual matrix *E*. The fundamental Equation ^29^ is shown by Eq. (1):

1 $${X}_{ij}={\sum }_{k=1}^{n}{g}_{ik}{f}_{kj}+{e}_{ij}$$

where *X*_*ij*_ is the concentration of species j in the ith sample, g_ik_ is the contribution of the kth source to the ith sample, f_kj_ is the distribution rate of species j in the kth source, and e_ij_ is the residual.

When operating the PMF program, the uncertainty is set to 5/6 of the detection limit when the concentration of the pollutant is below the detection limit and the uncertainty is set to^13^ when the concentration is above the detection limit^30^.

2 $${S}_{ij}=\sqrt{{(RSD\times {X}_{ij})}^{2}+{MDL}_{j}^{2}}$$

where S_ij_ is the uncertainty of species j in the ith sample, *X*_*ij*_ is the concentration of species j in the ith sample, RSD is the percentage error, and MDL_j_ is the method detection limit for species j.

#### Backward trajectory clustering and PSCF analysis

Trajectory clustering analysis was used to determine the trajectories of major pollution sources by clustering and grouping a large number of valid simulated trajectories using the similarity of the trajectory space^31^. The potential source contribution method is an analytical method based on airflow trajectory analysis and is used to determine the source areas of pollution affecting atmospheric quality^32^. This study used the backward trajectory model HYSPLIT^13^ to explore the sources and transport pathways for pollutants in Bengbu City from October, 2021 to September, 2022. The latitude and longitude of the sampling point were selected as the starting point. The study area was divided into a 0.5° × 0.5° grid and the Global Data Assimilation System (GDAS) data provided by the National Centers for Environmental Prediction were used to fit the 48 h backward trajectories at an altitude of 500 m, with daily simulated starting times of 00:00, 06:00, 12:00, and 18:00. All trajectories arriving at the starting point were cluster analyzed and the PSCF calculated using the Euler distance algorithm^13^.

#### Human health risk assessment

The benzo[a]pyrene (BaP) equivalent concentration (BaPeq) and inhalation lifetime cancer risk (ILCR)^33^ recommended by the US EPA were used to assess the hazard posed by PAHs to human health.

#### Carcinogenic risk

The BaPeq method uses the BaP concentration as a reference value to calculate the BaPeq and the total carcinogenic equivalent concentration (TEQ) of each PAH monomer, as shown in Eq. (3)^34^.

3 $$TEQ={\sum }_{i=1}^{n}\left({C}_{i}TEFi\right)$$

where TEQ is the total carcinogenic equivalent concentration of the PAHs; Ci is the biomass concentration of PAH monomers (ng/m^3^); TEFi is the toxic equivalency factor of PAH monomers, where the TEFs of NaP, Ace, Acy, Fl, Phe, Flu and Pyr are 0.001; Ant, BghiP, and Chry are 0.01; BaA, BbF, BkF, and InP are 0.1; and BaP and DahA are 1.

#### Incremental lifetime cancer risk

The ILCR due to PAHs from respiratory exposure was calculated using Eq. (4)^35^:

4 $$ILCR=CSF\times ADD$$

where ILCR is the lifetime carcinogenicity risk and CSF is the carcinogenicity strength factor (kg·day/mg) derived from animal data for humans. The value of CSF in the respiratory exposure pathway was set at 26.6, which is the maximum value of the assessed risk^35^. ADD is the average daily exposure dose (mg/(kg·day)) and was calculated according to Eq. (5):

5 $$ADD=\frac{CA\times IR\times ET\times EF\times ED}{{\text{BW}}\times {\text{AT}}}$$

where CA is the concentration of the contaminant in the air (mg/m^3^), IR is the respiration rate (m^3^/h), ET is the daily exposure frequency (h/day), EF is the annual exposure frequency (day/a), ED is the exposure duration (a), BW is body weight (kg), and AT is the average exposure time (day). The parameters of the exposure factor data^6^ are given in Table 2.

**Table 2:** Parameters for the exposure factor data.

Crowds	IR (m^3^/day)	ET (h/day)	EF (day/a)	ED (a)	BW (kg)	AT (day)
Adult	20	24	365	30	61	75 × 365
Children	7.5	24	365	10	16	75 × 365

## Results and discussion

### Characterization of PAHs contamination

#### Concentrations and seasonal trends

Over the study period, the ΣPAHs concentration in ambient air ranged from 1.71 to 43.85 ng/m^3^, with an annual mean mass concentration of 10.06 ± 8.04 ng/m^3^, of which Phe, Flt, Pyr, Chr, BbF, and BaP were the most abundant among the 16 PAHs, accounting for more than 60% of the total PAHs. The airborne total PAH concentrations recorded in this study were about 1/24 of those in the southeastern suburbs of Beijing^36^ (0.29–1184.48 ng/m^3^), 1/10 of those in the urban area of Xi’an City^37^ (35.79–472. 76 ng/m^3^), and 1/3 of those in Nepal^38^(4.30–131.00 ng/m^3^), but were about 1.5 times higher than Jinsha Town, Central China^39^ (11.21–30.75 ng/m^3^). This suggests that PAH pollution in suspended particulate matter in Bengbu City is at a medium level.

**Figure 1:**
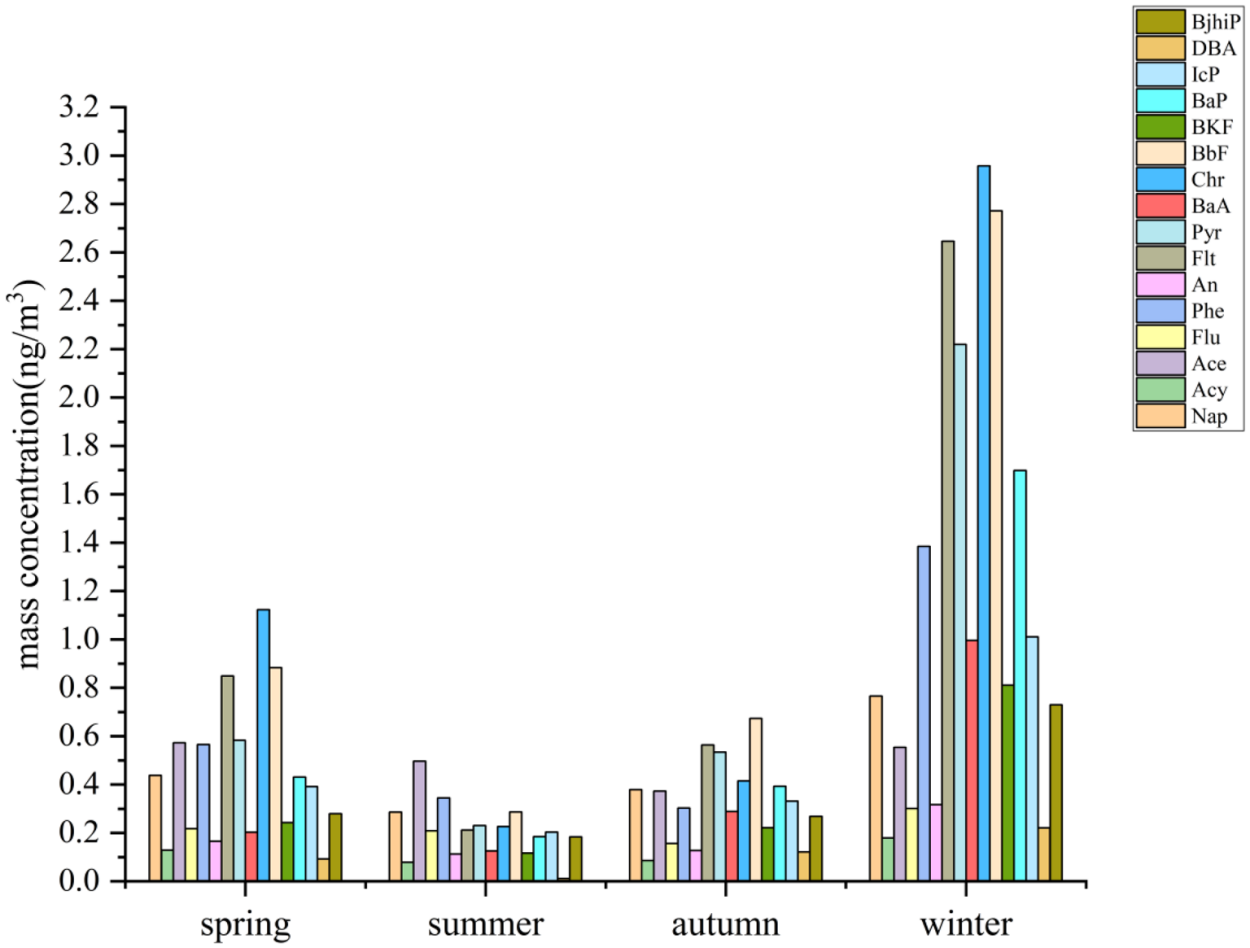
Mass concentrations of 16 PAHs in different seasons.

The PAH concentrations were significantly higher in winter (19.56 ± 7.80 ng/m^3^) than in the other three seasons (one-way ANOVA, *P* < 0.01), with no significant differences (*P* > 0.05) among spring (7.16 ± 5.77 ng/m^3^), summer (3.31 ± 1.05 ng/m^3^), and autumn (5.23 ± 2.84 ng/m^3^). The ρ(BaP) trend was winter (1.69 ng/m^3^) > spring (0.43 ng/m^3^) > autumn (0.39 ng/m^3^) > summer (0.18 ng/m^3^), which were all lower than the limit for the 24 h mean BaP concentration specified in the Ambient Air Quality Standard^2^ (GB 3095–2012) (2.50 ng/m^3^). Figure 1 shows that the results were consistent with the PAHs trend reported by Li et al^40^. in the West Third Ring area of Beijing, where the average concentrations of both ΣPAHs and BaP showed a seasonal trend of winter > spring > autumn > summer.

The reasons for these variations may be seasonal differences in emissions from pollution sources and meteorological conditions. The seasonal meteorological conditions during the sampling period are shown in Table 3 and it can be seen that there are clear differences in the meteorological conditions between summer and autumn in Bengbu City. In addition, there is frequent rainfall in summer, which can effectively disperse and settle the pollutants^41^, therefore, the PAHs pollution level in summer is lower. High mass concentrations of PAHs in winter are caused by the greater number of sources and emissions and by the meteorological conditions, which are less favorable for the dispersion of pollutants than in summer and are more suited to combustion related pollution^42^.

**Table 3:** Different meteorological conditions during the sampling period.

Season	Temperature/(℃)	Relative humidity/(%)	Barometric pressure/(hpa)	Wind velocity/(m s^−1^)	Rainfall intensity/(mm day^−1^)
Spring	17.3 ± 4.5	63.2 ± 3.7	1010.1 ± 2.5	2.8 ± 0.4	0.08 ± 0.04
Summer	28.8 ± 0.5	74.8 ± 5.5	996.4 ± 7.9	2.5 ± 0.4	1.29 ± 0.04
Autumn	15.9 ± 6.9	64.3 ± 6.9	992.9 ± 29.9	2.4 ± 0.3	0.24 ± 0.03
Winter	2.7 ± 2.4	66.7 ± 3.7	1023.9 ± 0.2	2.4 ± 0.6	0.23 ± 0.01

#### Distribution characteristics of PAHs with different ring numbers

The 16 PAHs can be divided into three groups: Nap, Ace, Acp, Flu, Phe, and Ant are 2 or 3 ring PAHs, i.e. low-ring PAHs, which are mainly present in the gas phase; Flt, Pyr, BaA, and Chry are 4 ring PAHs, i.e. PAHs with a medium ring count, which are present in both the solid and the gas phases; and BbF, BkF, BaP, Ind, DahA, and BghiP, which are 5 or 6 ring PAHs, i.e. PAHs with a high ring count and are mainly distributed in the solid phase of particulate matter^41^.

**Figure 2:**
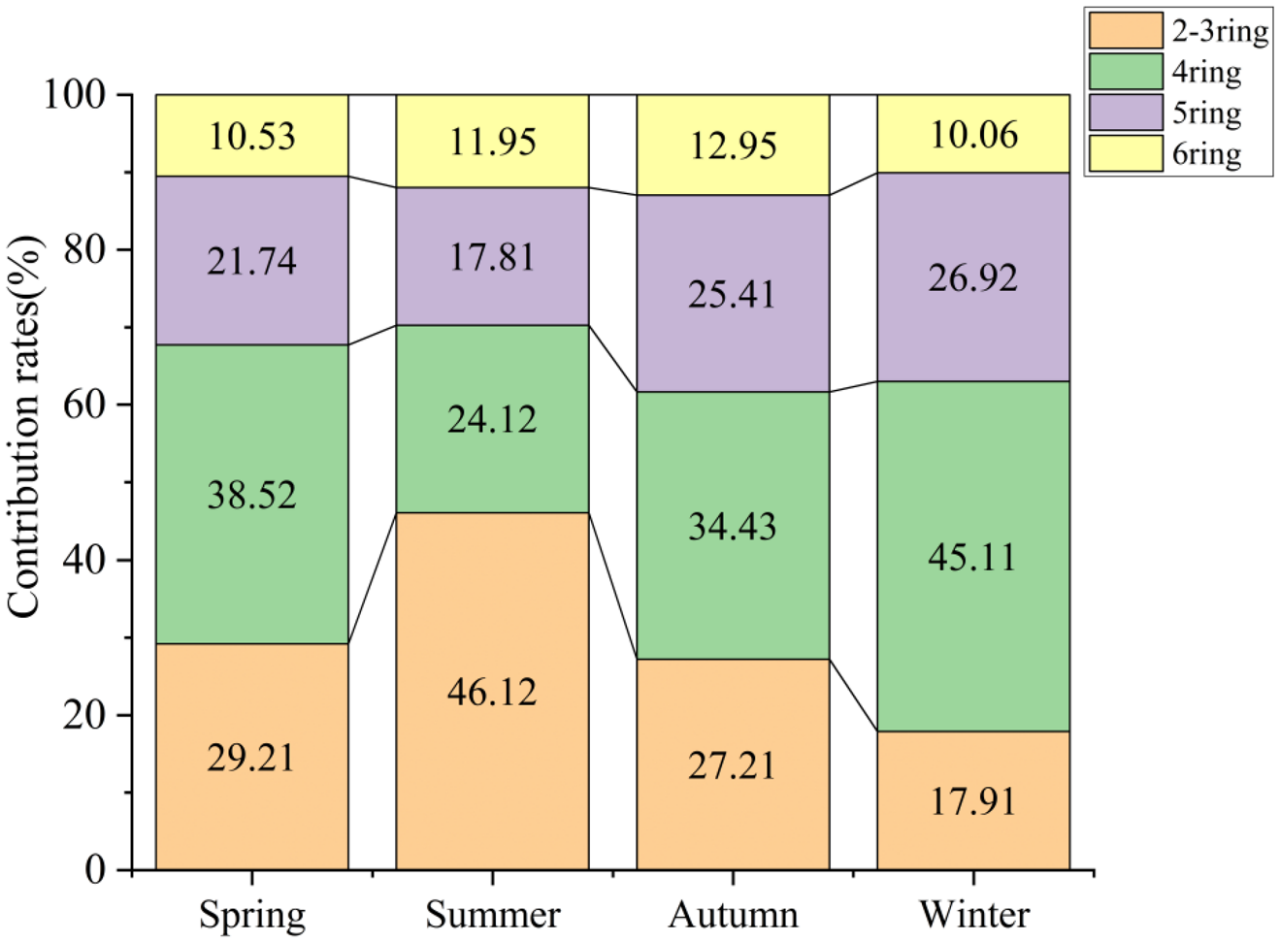
Contributions made by PAHs with different ring numbers over the four seasons.

The contributions made by PAHs with different ring numbers in different seasons are shown in Fig. 2 and the ΣPAH percentage order for the four seasons was winter (55.46%) > spring (20.31%) > autumn (14.84%) > summer (9.39%), which was consistent with those reported by Qiong Deng et al^43^. in the eastern suburb of Chengdu and Yan Wang et al^8^. in a suburb of Dalian. The annual ΣPAHs results show that the proportions of PAHs with different ring numbers were as follows: 4 rings (40.17%) > 5 rings (24.7%) > 2-3 rings (24.21%) > 6 rings (10.92%). Among the percentages for the ringed PAHs in the TPAHs, the values for the 4-5 ring PAHs were highest in spring, autumn, and winter, whereas during summer the 2-3 ringed PAHs were higher than those of the other PAHs and significantly higher than for all other seasons. The proportions of 4 ring and 5 ring PAHs were the largest throughout the year, accounting for 64.87% of the total, followed by 2-3 ring and 6 ring PAHs, accounting for 35.13% of the total, and this result was consistent with the results reported by Zou Zehao et al^44^. for cities in the Pearl River Delta. The reason for this result may be that the higher temperature in summer facilitates the conversion of PAHs from the particulate phase to the gas phase, while high ambient humidity and low temperatures in winter promote the conversion of PAHs from the gas phase to the particulate phase, resulting in higher concentrations of PAHs in winter compared to summer^45^.

### Source apportionment

#### Diagnostic ratios for PAHs

Most environmental samples contain PAHs from mixed sources and the diagnostic ratio method is widely used to identify the sources of the PAHs^10^. Calculations were performed to infer the sources based on the ratios and the results from this study are shown in Table 4.

**Table 4:** Diagnostic ratios for PAHs in Bengbu City for the different seasons.

Diagnostic ratio	Ratio range	Source	Ratio results			
			spring	Summer	Autumn	Winter
InP/(InP + BghiP)^46^	< 0.2	Oil source	0.61 ± 0.10	0.42 ± 0.02	0.54 ± 0.02	0.58 ± 0.02
	0.2–0.5	Liquid fossil fuel combustion				
	> 0.5	Coal and biomass combustion				
Flu/(Flu + Pyr)^14^	< 0.4	Oil source	0.54 ± 0.36	0.46 ± 0.09	0.26 ± 0.12	0.12 ± 0.06
	0.4–0.5	Liquid fossil fuel combustion				
	> 0.5	Coal and biomass combustion				
BaA/(BaA + Chr)^6^	< 0.2	Oil source	0.11 ± 0.09	0.45 ± 0.18	0.45 ± 0.07	0.25 ± 0.04
	0.2–0.35	Oil or combustion sources				
	> 0.35	Combustion source				
Ant / (Ant + Phe)^14^	< 0.1	Oil source	0.25 ± 0.05	0.27 ± 0.05	0.32 ± 0.09	0.19 ± 0.03
	> 0.1	Combustion source				

The InP/ (InP + BghiP) values for Bengbu were 0.53–0.69, 0.50–0.59, and 0.54–0.62 in spring, autumn, and summer, respectively, and the ratios were higher than 0.5, indicating that there were contributions from the coal and biomass combustion sectors. The Flu/ (Flu + Pyr) values for Bengbu City ranged from 0.07 to 0.56 and 0.05 to 0.32 in autumn and winter, respectively, with mean values less than 0.4, while the values for BaA/ (BaA + Chr) in spring ranged from 0.10 to 0.24 with a mean value less than 0.2, suggesting that petroleum sources made contributions in spring, autumn, and winter. The Flu/ (Flu + Pyr) and InP/ (InP + BghiP) values in summer were 0.29–0.69 and 0.35–0.48, respectively, with average values of 0.46 and 0.42, implying that the main source of PAHs in summer was the combustion of liquid fossil fuels. The mean Ant/ (Ant + Phe) value was greater than 0.1 in all four seasons, indicating that both liquid fossil fuel combustion and biomass combustion contributed to PAH pollution in Bengbu City during the study period.

The diagnostic ratio method results showed that coal combustion, biomass combustion, petroleum sources, and liquid fossil fuel combustion were the main sources of PAHs in the TSP from Bengbu City and that the PAH emission sources in Bengbu City differed among the four seasons. This result can be compared to the Belgian coastal region^7^, where the main source of PAHs is motor vehicle emissions from petrol and diesel combustion, whereas in Bengbu there is a wider range of PAH sources in the atmosphere. In contrast, the PAHs sources in Changzhou city^47^ and the Venice area, Italy^48^, are basically the same.

#### Positive matrix factorization

**Figure 3:**
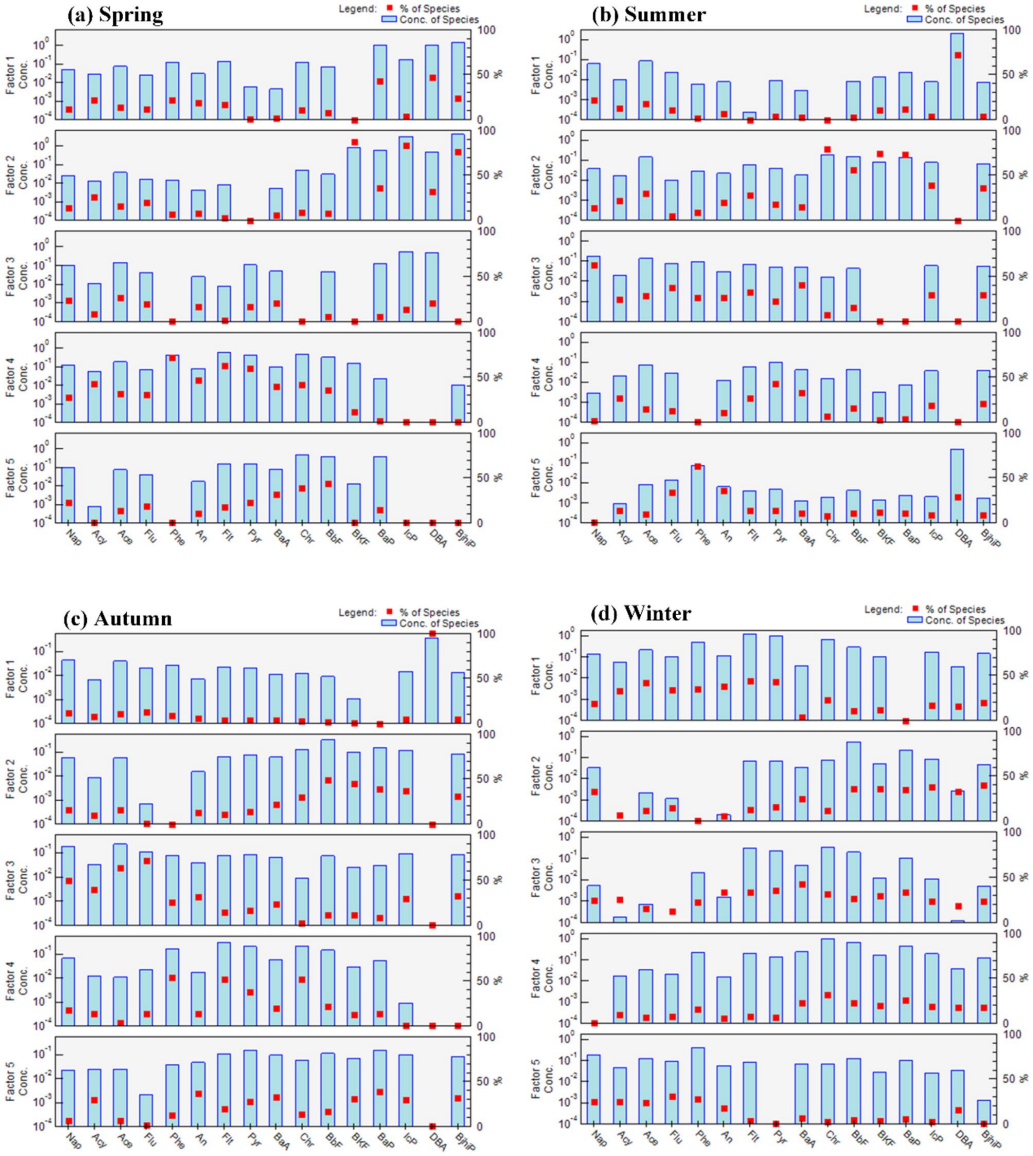
Source profiles and individual contributions made by each factor to the 16 PAHs species in the four seasons.

There were five different sources identified for the 16 UC–PAHs over the four seasons in Bengbu and the emission sources varied in each season, as shown in Fig. 3.

Fla, Pyr, Chry, and BbF are considered to be signature pollutants of coal combustion^49^. Therefore, Factor 1 in spring, Factor 1 in summer, Factor 5 in autumn, and Factor 1 in winter were considered to be sources derived from coal combustion, with the following four seasonal contributions: winter (37.6%) > autumn (36.9%) > spring (19.2%) > summer (6.9%). The contribution made by coal combustion is higher in autumn and winter than in spring and summer, probably due to increased heating demand and high coal consumption in autumn and winter^23^.

Ace, Acy, Flu, and Phe are considered to be typical contaminants produced by the volatilization of petroleum products and crude oil spills^50^, whereas Ace, Ant, and BaP are the principal pollutants produced by the coking industry^51^. Therefore Factor 4 in spring, Factor 5 in summer, Factor 3 in autumn, and Factor 5 in winter were considered to be industrial emission sources, with seasonal contributions as follows: spring (46.4%) > summer (35.8%) > autumn (31.5%) > winter (17.8%). This finding may be attributed to the greater contributions made by crude oil and petroleum volatile emissions due to higher temperatures in spring and summer, which meant that the contributions made by industrial emission sources were higher in spring and summer than in autumn and winter^10^.

The PAHs with high ring numbers, such as BaA, BbF, BkF, DahA, BghiP, and InP, are recognized as distinctive pollutants produced by petrol and diesel combustion. Therefore, Factor 2 in spring, Factor 2 in summer, Factor 2 in autumn, and Factor 2 in winter were considered to be traffic emission sources and made the following seasonal contributions: summer (20.5%) > autumn (12.7%) > spring (8.3%) > winter (5.9%). The large contribution made by petrol and diesel combustion sources in midsummer may be related to secondary reactions and the degradation of PAHs during the summer months when temperatures and light intensity are high.

Nap, Flu, BbF, and BkF are assumed to be specific components of biomass combustion, whereas Chry and BaA are produced by natural gas combustion^28^. Therefore, Factors 3 and 5 in spring, Factors 3 and 4 in summer, Factors 1 and 4 in autumn, and Factors 3 and 4 in winter are biomass versus natural gas combustion factors that made the following four seasonal contributions: winter (38.7%) > summer (36.9%) > spring (26%) > autumn (18.7%). Households are heated in winter and large quantities of fossil fuels are burnt^6^, whereas in summer, due to higher temperatures, volatile pollution from exhaust emissions by new energy vehicles based on natural gas is more severe^52^. Therefore, the contribution made by fossil fuels is the greatest in winter, followed by summer.

The results show that the dominant sources of PAH pollution in Bengbu in spring and summer are industrial emissions and petrol and diesel combustion, whereas the leading sources of pollution in autumn and winter are coal, biomass, and natural gas combustion, which is the same as results for the diagnostic ratio method. The main sources of PAHs in Bengbu were similar to 19 communities in Xi’an^6^, downtown Beijing^14^, and the Dushanzi area in Xinjiang^24^, which suggests that these areas are affected by the same or similar types of pollution sources.

#### Backward trajectory clustering with PSCF analysis

**Figure 4:**
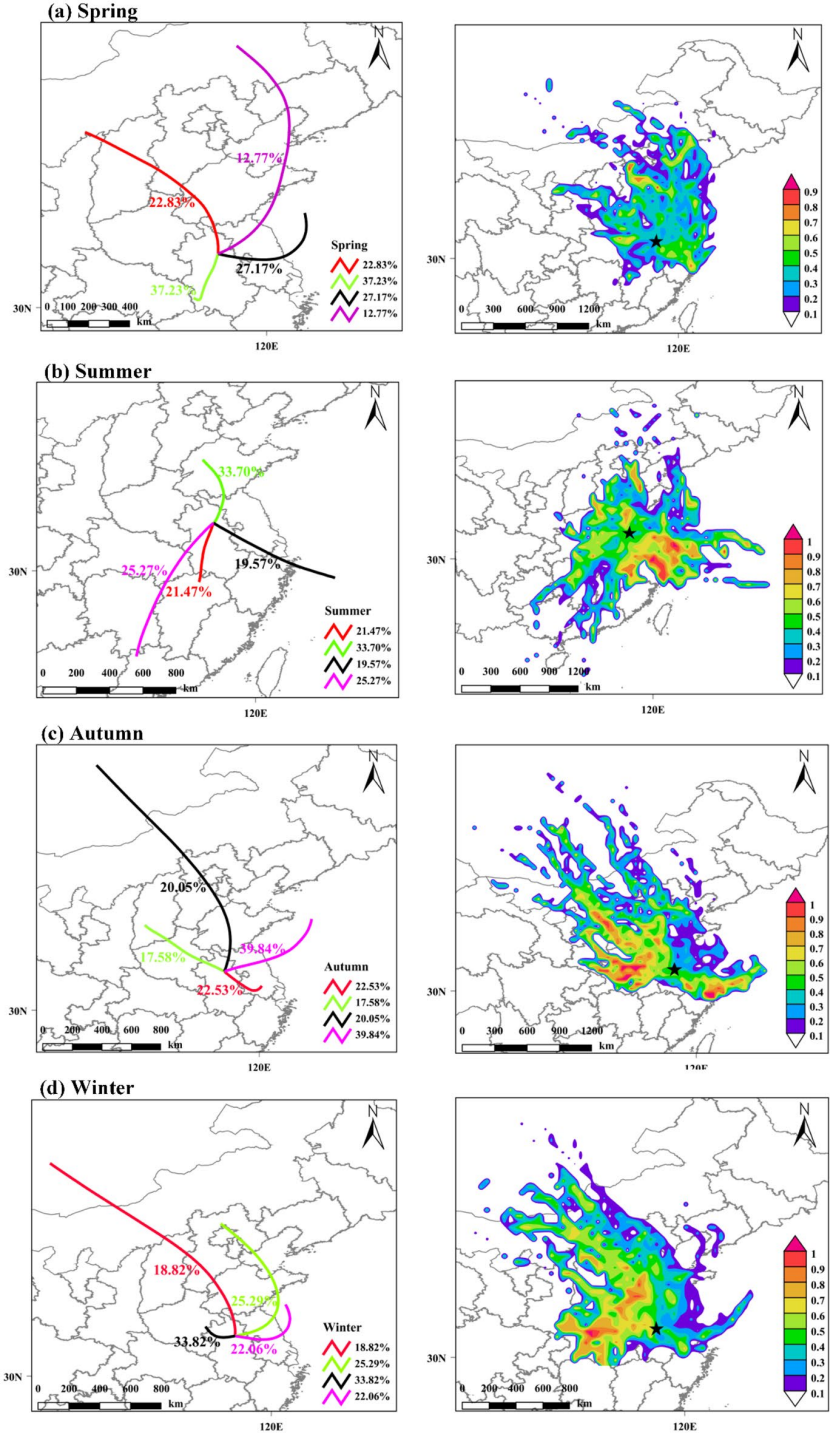
Backward trajectories and PSCF analyses for the different seasons.

The backward trajectories and potential sources during the sampling period in Bengbu were analyzed to further understand the sources of the PAHs in Bengbu. The backward trajectories can indicate the percentage of airflow from different regions, while the potential source contribution model (PSCF) further integrates the size of the percentage airflow and the concentration of pollutants it carries and calculates regional contributions to the concentration of target pollutants^13,23^. The results are shown in Fig. 4.

The backward trajectory during the spring sampling period was mainly influenced by cluster 1 (22.83%) from the northwest, cluster 2 (37.23%) from the southwest, cluster 3 (27.17%) from the eastern Yellow Sea, and cluster 4 (12.77%) from the northeast (Fig. 4a). The PSCF source simulation shows that the potential source has little effect on the PAHs concentration in Bengbu City. Hebei and Liaoning regions have a slightly greater influence on PAHs in Bengbu City, which is mainly influenced by a combination of air mass 1 (ρ ΣPAHs = 7.15 ng/m^3^) and air mass 4 (ρ ΣPAHs = 5.38 ng/m^3^). In addition, the concentration of PAHs in Bengbu City in spring was 7.165 ng/m^3^, which was similar to that of the air mass. The northern regions of China have frequent northerly winds in spring. These strong winds promote the long-range transport of air pollutants and may also promote the regional homogenization of pollutants. The Liaoning and Hebei regions, which are traditional industrial bases in China, make their largest industrial emission contributions to atmospheric PAHs in spring^10,30^ and these may partially increase the industrial emissions contributions to PAHs in Bengbu in spring.

The backward trajectory of the summer flow was mainly influenced by a combination of the southern air mass 1 (21.47%), northwestern air mass 2 (33.70%), southeastern air mass 3 (19.57%), and southwestern air mass 4 (25.27%) (Fig. 4b). The potential sources of pollution analysis in the summer PSCF showed that the potential sources were mainly located in the East China Sea and Zhejiang areas and were more affected by air mass 3 with an average pollutant concentration of 3.57 ng/m^3^, which slightly exceeded the average PAH concentration in Bengbu City (3.31 ng/m^3^). Coastal areas, such as Jiangsu, Zhejiang, and Shanghai, are among the most economically developed regions in China with high levels of car ownership. Studies in Shanghai, Nanjing, and Hangzhou have shown that traffic emissions are the largest contributors to summertime atmospheric PAHs in these areas with contribution rates as high as 34.5%, 45.8%, and 38.2%, respectively^13,20,53^. The proportion due to traffic emission sources in summer in Bengbu reached 20.5%, which was much higher than in the other three seasons (8.3%, 12.7%, and 5.9%, Fig. 3b). The input due to polluted air masses from coastal areas, such as Jiangsu, Zhejiang, and Shanghai, during the monsoon period may also be one of the reasons for the increase in the atmospheric PAHs proportion due to traffic emission sources in summer in Bengbu.

The backward trajectory of the autumn airflow mainly consisted of southeast air mass 1 (22.53%), west-northwest air mass 2 (17.58%), northwest air mass 3 (20.05%), and northeast air mass 4 (39.84%) (Fig. 4c). The autumn PSCF results for Bengbu showed that the potential sources of PAHs were mainly located in Henan, Hubei, Shaanxi, and Shanxi and were strongly influenced by a combination of air mass 2 (ρ ΣPAHs = 13.11 ng/m^3^) and air mass 3 (ρ ΣPAHs = 6.10 ng/m^3^). The autumn atmospheric concentration of PAHs in Bengbu was 5.235 ng/m^3^ and was influenced by long-range atmospheric transport. Shanxi and Shaanxi Provinces are rich in coal reserves and coal has long been a major energy source in these provinces. Coal use is also common in Henan and Hubei Provinces^15,30^. Coal combustion in Zhengzhou and Xi’an contributes 38.0% and 46.6% to atmospheric PAHs in autumn, respectively^5,53^. Therefore, the migration of the northwestern coal combustion pollution stream in autumn and winter may have exacerbated autumn PAH pollution in Bengbu City.

The backward trajectory of the winter airflow was mainly caused by northwestern air mass 1 (18.82%), northeastern air mass 2 (25.29%), western air mass 3 (33.82%), and eastern air mass 4 (22.06%) (Fig. 4d). The winter PSCF model results show that the potential sources of PAHs in winter were mainly concentrated in Shandong, Henan, Hubei and northern Hebei regions, and were mainly affected by the combined effect of air masses 1, 2, and 3. The average concentrations of PAHs in air masses 1, 2, and 3 were 25.08 ng/m^3^, 22.62 ng/m^3^ and 17.05 ng/m^3^, respectively, while the atmospheric concentration of PAHs in Bengbu in winter is 19.564 ng/m^3^. The large area north of the Huai River in China contains many centrally heated areas in winter where considerable amounts of coal are burnt. Bengbu is located by the Huai River but does not have many areas that are centrally heated in winter. Air masses 1 and 2 from the north may introduce coal-fired pollution streams into the city, further exacerbating PAH pollution levels in Bengbu and aggravating the contribution made by coal combustion sources. Air mass 3 is mainly from Hubei Province, west of Anhui Province, which is also a non-central heating area where many people heat their homes by burning coal or straw from the autumn harvest. Zhang Y et al^54^. showed that the main sources of ambient PAHs in Wuhan, Hubei Province, during the autumn and winter seasons were coal and biomass combustion (54.3 ± 11.3%) and their long-range transport may also partially alter the concentration and contribution ratio for PAHs in Bengbu.

The spatial analysis of potential sources of pollution in Bengbu based on the backward trajectory model showed that the pollution in spring, autumn, and winter was mainly caused by the northern and northwestern regions of China, with the northern region being more important in winter and the coastal region south of Bengbu being the most important in summer, which basically agrees with the atmospheric PAH results for Shanghai^55^ and Wuhan^54^, both of which are close to Bengbu.

### Health risk assessment

#### Exposure assessment

The TEQ is widely used to measure the carcinogenic risk of each PAH. Among the 16 PAHs selected for priority control, the BaP content roughly mirrors the contamination status of ∑PAHs in ambient particulate matter. This allows the other categories of PAHs to be converted into equivalent concentrations based on BaP and is known as the Toxic Equivalent Factor (TEF)^35^.

The total annual carcinogenic equivalent concentration values for PAHs due to respiratory exposure ranged from 0.0159–7.437 ng/m^3^. The average annual carcinogenic equivalent content in Bengbu was lower than those^56^ for Harbin (46.00 ng/m^3^), Wuhan (141.36 ng/m^3^), and Nanjing (289.69 ng/m^3^). However, it was higher than that for Shenzhen (2.05 ng/m^3^), which suggested that the carcinogenic equivalent concentration of PAH pollution in Bengbu was moderately high. The results showed that TEQ winter (3.406 ng/m^3^) > TEQ autumn (1.160 ng/m^3^) > TEQ spring (1.082 ng/m^3^) > TEQ summer (0.325 ng/m^3^). The TEQ values were above the WHO limit value (1 ng/m^3^)^57^ in spring, autumn, and winter and were only below the limit value in summer, which suggests that there are different potential carcinogenic risk levels in spring, autumn, and winter.

#### Incremental lifetime cancer risk (ILCR)

The US EPA classifies cancer risk as acceptable risk (ILCR < 10^–6^), potential cancer risk (10^–6^ < ILCR < 10^–4^), and higher cancer risk (10^–4^ < ILCR), where an increased ILCR value indicates a greater cancer risk^58^.

The ILCR values for PAHs produced by the respiratory exposure pathway in Bengbu City for the different seasons are shown in Table 4, where annual ILCRs ranged from 1.431 × 10^–4^ to 3.671 × 10^–3^ for adults and from 6.823 × 10^–5^ to 1.749 × 10^–3^ for children. The lifetime exposure risk to PAHs for adults and children was greater than 10^–4^ throughout the year and in each of the seasons, implying a high carcinogenic risk from PAHs throughout the year. Furthermore, the values were much higher than the urban ILCR values for northern Thailand^59^ (6.80 × 10^–5^) and Mianyang, Sichuan Province^60^ (1.55 × 10^–5^), China, which suggested that there was a substantial health risk from PAHs in Bengbu City.

The lifetime risk of cancer for the four seasons was as follows for both adults and children: ILCR winter > ILCR spring > ILCR autumn > ILCR summer, as shown in Table 5. The risk of cancer was greatest in winter for both adults and children. The ILCR values for adults in winter were about three times higher than in spring, four times higher than in autumn, and six times higher than in summer, whereas for children the risk in winter was about 2.5 times greater than in spring, four times greater than in autumn, and five times greater than in summer, which is broadly similar to the health risk assessment for PAHs in Chengde City^10^. Adults have a lifetime carcinogenic risk of about two to three times that of children, probably because adults have a faster breathing rate and a greater exposure area than children^5^. The above results show that the potential cancer risk in Bengbu City is more severe in spring, autumn, and winter than in summer and that the pollution hazard is more significant in winter, where there is a stronger cancer risk for both adults and children, but the risk to adults is more serious Table 5.

**Table 5:** Lifetime cancer risk values for each season in Bengbu City.

Form	Spring ILCR	Summer ILCR	Autumn ILCR	Winter ILCR	Year-round ILCR
Adult	5.996 × 10^−4^	2.772 × 10^−4^	4.378 × 10^−4^	1.638 × 10^−3^	7.4 × 10^−4^
Children	2.857 × 10^−4^	1.319 × 10^−4^	2.085 × 10^−4^	7.804 × 10^−4^	3.5 × 10^−4^

**Figure 5:**
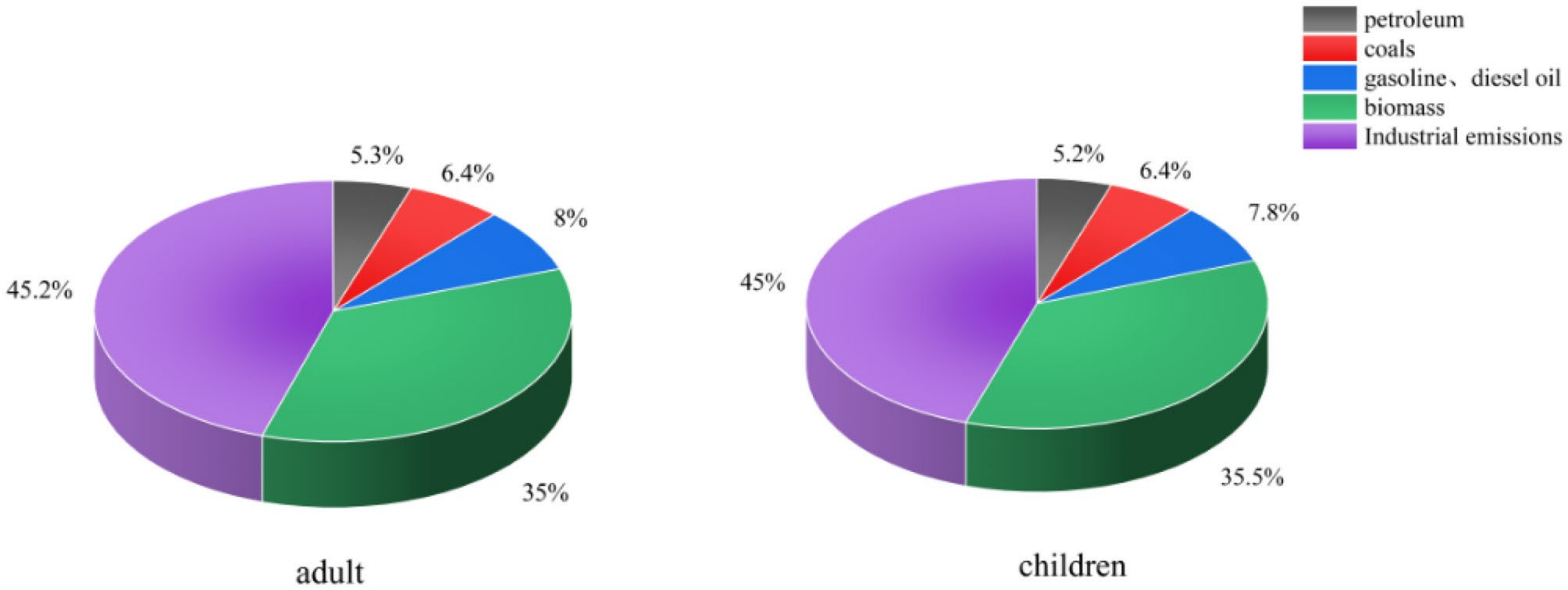
Annual health risks from five PAH sources for children and adults.

The ILCR due to the five emission sources for adults and children throughout the year was calculated by combining the emissions from different sources, as shown in Fig. 5. For adults, combustion sources, such as biomass, natural gas, and coal, had the largest impact (4.2 × 10^–6^), representing about 46.7% of the total, followed by industrial emission sources (4.0 × 10^–6^), which accounted for 45.2% of the total, and finally petrol and diesel combustion sources (7.1 × 10^–7^), which accounted for about 8% of the total. The impact of the five sources on children throughout the year was close to that for adults, with fossil fuel combustion sources being the highest ILCR (2.0 × 10^–6^), corresponding to 47.1% of the total, followed by industrial emissions (1.9 × 10^–6^), corresponding to about 45% of the total, and finally petrol and diesel combustion sources (3.3 × 10^–7^) at about 7.8%. Over the year, the ILCR from combustion emissions, such as coal burning and industrial emissions, was relatively high. Therefore, the authorities should take measures to control exposure to PAHs from combustion sources and industrial emissions to reduce the health risks to adults and children.

## Conclusions

The TSP range for ρ(PAHs) in Bengbu City was 1.71–43.85 ng/m^3^ and the annual mean mass concentration was 10.064 ± 8.047 ng/m^3^, with the mean concentrations of both ΣPAHs and BaP showing a seasonal trend of winter > spring > autumn > summer. These seasonal variations were possibly caused by changes in meteorological factors. In terms of the number of rings, 4–5 ring PAHs accounted for the largest proportion in spring, autumn, and winter, whereas 2–3 ring PAHs were the largest proportion in summer. The diagnostic ratio method and PMF analysis results revealed that the sources of PAHs in Bengbu City varied among the four seasons. In spring and summer, industrial emissions and gasoline and diesel combustion were the primary sources of PAHs pollution, while in autumn and winter, coal, biomass, and natural gas combustion were the main sources of pollution. The backward trajectory and potential source analyses showed that the northern and north-western regions of China were the main sources of the pollution in spring, autumn, and winter, especially the northern region in winter, whereas the coastal region south of Bengbu was more important in summer. The results also showed that the PAH emissions from individual regions affect the Bengbu region through atmospheric and airborne particulate matter transport.

The health risk estimation showed that the total carcinogenic equivalent concentration values for PAHs in Bengbu City were between 0.0159 and 7.437 ng/m^3^ over the whole year, which is classified as moderate; the lifetime carcinogenic risk due to PAHs for adults and children exceeded the standard limit values in all seasons; and the ILCR level for adults was about 2–3 times higher than that for children. The ILCR order for the adult and child populations during the sampling period were winter > spring > autumn > summer, which suggests that the potential cancer risk varies across the spring, autumn, and winter seasons and that the health risks are significantly greater in winter.

## Data Availability

All data generated or analyzed during this study are included in this published article.
